# Decoding key aroma components of three cigar tobacco leaves based on molecular sensory science

**DOI:** 10.3389/fchem.2025.1633850

**Published:** 2025-07-10

**Authors:** Zhang Xiaowei, Wang Shuqi, Zhang Ke, Feng Liang, Xu Anchuan, Zhong Risheng, Chen Dan, Wang Chunqiong, Zhang Jiwu, Wan Yueying, Long Jie, Chen Haitao

**Affiliations:** ^1^ Yunnan Tobacco Quality Inspection and Supervision Station, Kunming, Yunnan, China; ^2^ Beijing Key Laboratory of Flavor Chemistry, Beijing Technology and Business University, Beijing, China; ^3^ Technology Center, China Tobacco Yunnan Industrial Co., Ltd., Kunming, Yunnan, China; ^4^ Honghe Prefecture Company of Yunnan Tobacco Company, Honghe, Yunnan, China

**Keywords:** cigar, key aroma components, gas chromatography-olfactometry-mass spectrometry, molecular sensory science, aroma recombination

## Abstract

Cigar is a flavor-dependent cash crop. However, the key aroma compounds of tobacco leaves are less studied. In this study, we used molecular sensory science to explore the key aroma compounds of cigar tobacco leaves from three different origins in Yunnan, China. The results showed that a total of 33 aroma compounds were quantitatively analyzed in the three tobaccos, among which there were eight key aroma components in YXYY, eight in DHYY, and four in PEYY with odor activity value (OAV)≥1 and flavor dilution (FD)≥2. Through recombination and omission experiments, the key aroma actives were further identified as phytol, acetic acid, isovaleric acid, 3-methylpentanoic acid, and (E)-5-isopropyl-8-methylnona-6,8-dien-2-one in YXYY, styrene, (E)-5-isopropyl-8-methylnona-6,8-dien-2-one, irisone, and phytol in DHYY, and acetic acid, styrene, and phytol in PEYY. In conclusion the present study revealed the key aroma compounds and their differences in cigar tobacco from three different origins. It provides insights for a comprehensive exploration of the unique flavors of cigars.

## 1 Introduction

Cigar is a tobacco product other than traditional cigarettes, usually a smokable roll made from fresh cigar tobacco leaves that have been dried, fermented, matured, and hand-rolled (Fan et al., 2023). A cigar consists of three layers from inside to outside, which are, in order, the filler, the binder and the wrapper, and is also known as the core, inner binder and outer wrapper tobaccos in our country. As the outermost layer of tobacco, the wrapper is required to have good combustibility, uniform color and vein density; the main role of the binder is to fix the core, which requires the selection of tobacco varieties with high mechanical strength; the filler mainly assumes the function of combustion regulation and flavor release, which requires the use of tobacco to have good combustibility and outstanding style of aroma characteristics (Zhang M. Z. et al., 2024). Cigars are enjoyed by many domestic and international consumers for their unique flavor, strength, rich and delicate aroma, bitter-sweet taste and experience (Deng, 2021). In recent years, China’s economic level has been rising, the middle and high income groups have further expanded, and the domestic cigar sales market has developed strongly, with sales of domestic medium- and high-end handmade cigars exceeding 22 million sticks in 2021, realizing 100% growth year-on-year. The market potential of cigar cigarettes is getting bigger and bigger, and the growth momentum is strong (Zhu et al., 2024). China’s tobacco industry enterprises have also launched products with market competitiveness, including Hubei China Tobacco’s Huanghelou and Maoda cigars, Anhui China Tobacco’s Wangguan cigars, Sichuan China Tobacco’s Changcheng Cigar Series, and Shandong China Tobacco’s Taishan and Jiangjun cigars, which are representative of their products. However, from the perspective of the overall development of the industry, China’s cigar development is still facing a double challenge, on the one hand, the overall cigar market size is relatively small, the market potential to be further explored. On the other hand, the supply of high-end cigar raw materials is still dominated by imports, and there is an obvious gap between the quality of domestic cigar raw materials and imported products, which directly restricts the process of industrial upgrading. Therefore, to realize the quality of cigar raw materials to improve the local cigar industry has become the core of the urgent need to break through the subject.

The sensory quality of tobacco raw materials directly determines the aroma style of the entire cigar tobacco product, and the growth of cigar tobacco leaves on the temperature, rainfall, soil and cultivation technology requirements are very high, so the world can grow high-quality tobacco is very limited in the region, mainly distributed in the North-South Tropic of Cancer, especially in the 23 °N latitude band to form a continuous production area belt, covering the traditional planting areas of Cuba, Dominican Republic, Honduras, etc., by virtue of the unique geographic advantages constitute the core production area of cigar raw materials (Zheng et al., 2022). For example, Cuba, known as the “capital of cigars,” is recognized as the origin of top-quality cigar raw materials due to its unique climate, mineral-rich soil and mature planting technology system, which cultivates tobacco materials with rich aroma and pure taste. The Honduras production area in Central America, on the other hand, through differentiated development, has formed a tobacco category known for its strong taste and distinctive spicy characteristics. The Dominican Republic is the largest producer of cigarillo tobacco, with a similar geographic location to Cuba and little difference in rainfall, and is known for its smoothness and mildness. The quality of wrapper cultivated in the Connecticut production area of the United States is very excellent, its tobacco has a wide leaf shape and excellent toughness, brown color, both the release of aroma and the sensory experience of the double advantages of rich layers, especially for the production of wrappers, and thus become the preferred raw material for high-end cigar products (Deng, 2021). Some other countries also produce cigar tobacco with their own characteristics.

China has a long history of traditional drying tobacco cultivation. The history of the introduction of cigar tobacco into China can be traced back to more than a hundred years ago, but the real introduction of cigar tobacco varieties cultivation has only been more than 20 years, the majority of varieties grown for foreign varieties or local drying tobacco varieties (Zhong, 2023). At present, cigar tobacco is grown in Sichuan, Hubei, Anhui, Hainan, Yunnan, Guangxi, Guizhou, Henan and other places, typically in Shifang City in Sichuan, Danzhou in Hainan, Yuxi in Yunnan, and Danjiangkou in Hubei. Among them, Yunnan Province, located around 23 °N, has similar latitude and similar climatic conditions compared with the world’s high-quality cigar tobacco production areas, with abundant rainfall, abundant sunshine, good temperature and humidity harmonization, and fertile red loam and sandy loam soils, which possesses a great potential for the development of international high-quality cigar tobacco, and it is a golden area that can not be missed (Tie et al., 2024). At present, Yunnan tobacco team completed thousands of agronomic traits phenome determination and pest detection analysis, tens of thousands of molecular tests, in the core germplasm resource base for germplasm resources precision grading, preferred varieties screening, independent selection and breeding of Yunxue No. 1, No. 2 and other series of high-quality varieties, to crack the independent supply of raw materials for domestic cigars, independent guarantee on the breakthrough progress (Du et al., 2022).

Due to the different varieties of production raw materials and processing technology, the aroma style of cigar tobacco leaves has obvious regional characteristics, and the aroma composition is an important factor in determining the sensory style characteristics of tobacco, which is closely related to the aroma quality of tobacco (Zhu et al., 2024). Therefore, the differences in the aroma components of cigar tobacco leaves from different origins were analyzed from the level of the composition of aroma compounds, so as to objectively evaluate the aroma quality of cigar tobacco leaves from different regions and clarify the aroma style characteristics of cigar tobacco leaves from different production areas. In recent years, domestic and foreign scholars have analyzed the aroma components of cigar tobacco leaves from different regions with the help of different extraction methods and assays, such as solvent-assisted evaporative extraction (SAFE), solid-phase microextraction (SPME), simultaneous distillation extraction (SDE), gas chromatography-mass spectrometry (GC-MS), gas chromatography-olfactometry-mass spectrometry (GC-O-MS), gas chromatography-ion mobility chromatography (GC-IMS), and so on, with the aim of analyzing the aroma components of cigar tobacco leaves from different regions with the help of different extraction methods and assays, such as volatile compounds and key aroma active compounds. Compounds and key aroma active compounds as the focus of research to analyze the material composition of sensory differences between different cigar tobacco leaves. Jian Wang et al. (Wang et al., 2023) used GC-IMS to study and compare the volatile compositions of eight types of cigarillo filler tobaccos, and identified a total of 93 volatile compounds, including 12 esters, 4 olefins, 17 ketones, 4 acids, 20 aldehydes, 13 alcohols, 3 sulfur-containing compounds, 10 nitrogen-containing compounds, and 10 other classes. Shi et al. (2023) identified 82 volatile compounds from cigar tobacco by using static headspace/gas chromatography-ion mobility spectrometry coupled with multivariate statistical analysis, and 11 types of differential markers such as trimethylamine (ichthyic), ethyl 3-methylbutanoate (fruity), and 2,3-butanedione (buttery, creamy) were identified based on OAV. In addition, electronic nose (E-nose) and principal component analysis (PCA) were used to quickly differentiate the aroma profiles of different cigar varieties. Zhou et al. (2024) analyzed the volatile aroma compounds of six different varieties of Great Wall cigars using methods such as HS-GC-IMS and E-nose, and identified a total of 88 compounds, of which 24 compounds, such as 2-heptanone, n-butanol, 2,6-dimethylpyrazine, and 2-furfurylmethylsulfide, were considered as the major differential constituents, and nitrogen-containing compounds (such as pyrazine, trimethylamine) were identified as the main source of the irritating ammonia flavor and baking aroma of cigars. At present, there are fewer studies on the aroma composition of cigar tobacco in China. It is reported that the number of journal articles on cigar tobacco is only about 1/100 of that on conventional cigarettes, and there are even fewer articles on the chemical composition of cigar tobacco (Zhou et al., 2024). Moreover, the sensory style characteristics of cigar tobacco in various production regions are different, and the quality characteristics are obvious, and there is a lack of systematic and in-depth knowledge about the sensory and flavor composition of different varieties of cigar tobacco in different regions.

Molecular Sensory Science, as an important branch of modern food science, is a research method to systematically analyze the sensory properties of food through multidisciplinary cross technology. This theory was first proposed by Prof. Peter Schieberle of the Technical University of Munich (TUM) in 2006, and its core objective is to integrate the theories and technologies in the fields of analytical chemistry, sensory evaluation, and neurobiology, to reveal the chemical nature of food flavor formation at the molecular level, and to construct a recombinant model that can accurately simulate the sensory characteristics of natural food. The technical framework usually starts with the screening of key flavor compounds, and systematically identifies active ingredients with significant sensory contributions in food products through advanced analytical tools such as gas chromatography-olfactometry (GC-O), aroma extract dilution analysis (AEDA), and stable isotope dilution analysis (SIDA), in combination with sensory threshold measurements. On this basis, the researchers constructed an aroma recombination model and carried out deletion experiments to verify the necessity of each compound to the overall flavor, and finally realized the breakthrough of reproducing the sensory characteristics of complex foods with a small number of key molecules. The scientific value of molecular sensory science is not only reflected in its revelation of the mapping relationship between sensory attributes and chemical composition of food products, but also through the establishment of the quantitative correlation model of “ingredient-sensory-functionality”, which promotes the transformation of the food industry from empirical to data-driven, and provides a key technological pathway to the development of personalized flavor design, precise control of processing and health-oriented food. It provides a key technology path for personalized flavor design, precise control of processing and health-oriented food research and development. In this study, the key aroma compounds of three kinds of cigar tobacco in Yunnan were revealed by molecular sensory science, which provides a certain data foundation for the long-term and high-quality development of the local cigar industry.

## 2 Materials and methods

### 2.1 Samples

The cigar tobacco samples were provided by Yunnan Tobacco Quality Supervision and Inspection Station, and the samples were selected from Yuxi Yunxue No. 6 central cigar core tobacco (YXYY), Dehong Mangshi Yunxue No. 36 central cigar coated tobacco (DHYY), and Pu’er Yunxue No. 2 central cigar coated tobacco (PEYY) in Yunnan Province. In addition, the samples were subjected to the same conditions of grade, cultivation, drying and fermentation. All the samples were equilibrated in a constant temperature and humidity box (20°C, 70%–75% humidity) for 48 h, and stored for reserve.

### 2.2 Chemicals

The liquid nitrogen, high purity helium (purity ≥99.999%) and high purity nitrogen (purity ≥99.999%) used in this experiment were purchased from Beijing Ruizhi Hanxing Technology Co. Dichloromethane and anhydrous sodium sulfate were of analytical grade from Sinopharm Chemical Reagent Co. N-alkane mixtures (C_6_-C_30_) were obtained from Supelco, 2-Methyl-3-heptanone (98%), 2-Octanol (99%), 3-Acetylpyridine (99%), Acetophenone (99%), Neoprene (90%) were obtained from Aladdin. Styrene (99.5%) was obtained from Innochem. Phytol (≥90%) was obtained from Psaitong. β-Nicotyrine (98%), Irisone (97.89%) were obtained from TARGETMOL. Dihydroactinidiolide (99.7%), cedrol (99.8%), 2,3′-bipyridine (99.1%), 6-methyl-5-hepten-2-one (97%), myosmine (98%), 2-ethylhexyl acetate (99.6%) were obtained from TMRM Quality Inspection Technology Co. 5,6-Dihydro-6-pentyl-2H-pyran-2-yl acetate (99.6%) was obtained from TANMO. 5,6-Dihydro-6-pentyl-2H-pyran-2-one (90%), phytonadione (98%) were obtained from the Shanghai yuanye Bio-Technology Co., Ltd. 2,3-Dimethylpyrazine (99%), acetic acid (99.7%), isovaleric acid (98%), N-methylpyrrolidone (99%), 3-methylpentanoic acid (98.5%), acetamide (99%), phenylethanol (98.5%), 2-pyrrolidone (98%), indole (99%), benzaldehyde (98%), 2,3-butanediol (99%), phenylacetic acid (99%), 2,6-dimethylpyrazine (98%) were obtained from J&K Scientific Co.

### 2.3 Sensory evaluation

The experiment was conducted using a standardized sensory evaluation procedure. The experiment was carried out in a dedicated laboratory for sensory analysis in a controlled environment (25°C ± 1°C), 19 professionally screened sensory evaluators (9 males and 10 females, with an age structure of 20–28 years old and without rhinitis) was assembled. All members were experienced in sensory evaluation and had completed a 14-day training on cigar tobacco aroma characterization to ensure that they were able to accurately describe the aroma characteristics of cigar tobacco and accurately construct a sensory description system (Zhang J. et al., 2024). The first round of evaluation focused on the construction of flavor profiles, using a standardized sample preparation process (5 g/sample encapsulation). Each member of the team completed the sniffing and independently recorded the sensory descriptors of the cigar tobaccos. After counting, discussing and screening, eight descriptors for the aroma profiles of cigar tobacco were identified, namely: hay, sour, fermented, fruity, sweet, floral, burnt, and woody. In the second round, the sensory response strengths of the identified sensory descriptors were evaluated using the 0–10 scale method, 0 (no sensory state), 1–2 (critical sensory strength), 3–4 (weak sensory strength), 5–6 (steady state sensory strength), 78 (significant sensory strength), and 9–10 (suprathreshold sensory strength). Each evaluator performed three independent sniffing tests, recorded the instantaneous perceptual scores, and finally took the arithmetic mean as the characteristic aroma intensity index.

### 2.4 Aroma extraction by solvent assisted flavor evaporation

10 g cigar tobacco leaves were crushed into minced tobacco and put into a conical flask, 100 mL of redistilled dichloromethane was added as the extraction solvent, and 50 μL of 2-octanol solution (1.26 μg/μL), 50 μL of 2-methyl-3-heptanone (concentration of 1 μg/μL) were added as the internal standard. After sealing, the extract was continuously extracted for 1 h at room temperature with a thermostatic magnetic stirrer (1000 r/min) and vacuum filtration was performed. The filtrate residue was collected and reintroduced into 100 mL of redistilled dichloromethane to repeat the above steps, and the two extracts were combined.

The extracts were introduced into solvent assisted flavor evaporation (SAFE), and the phase separation of volatile components was completed under ultra-high vacuum (10^−5^ mbar). The obtained fractions were dehydrated by anhydrous sodium sulfate, further concentrated to 1.5 mL by Vigreux fractionator (50 cm × 1 cm) and filtered by microporous membrane, and then concentrated to 1 mL by nitrogen purging technique. The samples were sealed and stored in an ultra-low-temperature refrigerator at −40°C for subsequent analysis.

### 2.5 GC-O-MS analysis

The separation of volatile components was achieved on a TG-WAX (30 m × 0.25 mm×0.25 μm, 30 m) column, and the mass spectrometry detector was synchronously connected to the olfactory detection port through a Y-shaped splitter (split ratio 1:1). Each sample was recorded by three trained sensory evaluators. The temperature gradient program of the GC column temperature chamber was as follows: an initial temperature of 50°C, a constant temperature of 2 min, an increase in temperature to 120°C at 6 °C/min for 4 min, an increase in temperature to 200°C at 4°C/min, and an increase in temperature to the final temperature of 240°C at 8°C/min for 8 min. Ultra-high purity helium (99.99%) was used as the carrier gas, and 1.0 mL/min was used as the carrier gas. The mass spectrometry conditions were as follows: EI ionization source, ionization energy of 70 eV, ion source temperature of 250°C, mass range of 40–550 m/z, full scan, solvent delay of 3.5 min.

### 2.6 Aroma extraction dilution analysis

The aroma extraction dilution analysis (AEDA)was used to determine the aroma intensity of the characteristic aroma substances and to obtain the flavor dilution (FD) through the GC-O-MS platform in order to identify the important aroma active compounds in cigar tobacco. The flavor-enriched extract was diluted in a geometric gradient (dilution gradient of 1:2n) using dichloromethane as the dilution medium. Subsequently, the olfactory threshold was determined under the same GC-O-MS analytical conditions, and the dilution was terminated when no aroma was detected by three consecutive sniffing tests, at which time the FD value of the compound was determined to be the maximum dilution at which the substance could be smelled. In order to eliminate individual differences in olfactory sensitivity and to ensure the accuracy of the experiments. Each dilution gradient was sniffed by three trained sensory evaluators (one male and two female). The validity of the dilution gradient was confirmed only when all members perceived the target aroma simultaneously.

### 2.7 Qualitative analysis of aroma compounds

The qualitative analysis of aroma compounds was performed by similarity analysis based on NIST14 Library database with Xcalibur software data processing. The linear retention index (RI) of the aroma compounds were determined on a DB-WAX column and compared with the reference values (allowable deviation±20) (Acampora Zellner et al., 2008). Odor descriptions were matched with purchased standard compounds. The consistency of the chromatographic retention behavior of the target and the standard was compared under the same analytical conditions of GC-MS. The Retention Index (RI) was calculated from the retention times of a series of n-alkanes (C_6_-C_30_) in GC-MS by the following formula:

$$\text{RI}=100\times \left[n+\frac{\log\;t^{\prime }\left(i\right)-\log\;t^{\prime }\left(n\right)}{\log\;t^{\prime }\left(n+1\right)-\log\;t^{\prime }\left(n\right)}\right]$$

Where t'(i) is retention time of unknown compound. t'(n) and t' (n+1)-retention time of n-alkanes with n and n+1 carbon atoms.

### 2.8 Odor activity value

The odor activity value (OAV) quantification model was used to evaluate the contribution of the characteristic aroma components (Guadagni et al., 1966). When the OAV of an aroma compound was ≥1, it was determined that it contributed to the overall aroma of the sample. In this experiment, the sensory perception threshold value in the reference aqueous system was used as the benchmark (Gemert, 2011).

The OAV was computed using the following equation:

$$\text{OAV}=\frac{Ci}{OTi}$$

Where Ci is compound concentration and OTi is aroma threshold of the compound.

### 2.9 aroma recombination and omission

Aroma reconstitution: Key aroma components in the tobacco were screened based on sensory activity value (OAV≥1). They were mixed according to the accurately quantified concentrations and added to odorless glass bottles containing cellulose powder, and equilibrated at room temperature for 3 h. Subsequently, sensory evaluations were performed to compare the aromas of the recombinant model with those of the original tobacco samples, and to determine the intensities of the eight odor attributes. The average scores were calculated and the flavor radar charts were plotted.

Aroma omission: by sequentially missing an aroma compound from a reconstituted model. Multiple missing models were prepared and subsequently triangulated. Three brown glass bottles (including two full models and one missing model) were coded with three digits and presented to the sensory evaluator in a randomized order, who was asked to choose a different one based on its overall aroma profile. The results were expressed in terms of significance: highly significant difference (≥10 identifiers, α ≤ 0.001), highly significant difference (≥9 identifiers, α ≤ 0.01), and significant difference (≥8 identifiers, α ≤ 0.05), which further validated the key aroma-active compounds in tobacco.

### 2.10 Statistical analysis

Three parallel experiments were conducted and the results were expressed as mean ± standard deviation. The dataset of compounds was processed and analyzed using Excel version 2019 for visualization. Origin2021 software was utilized to express the results as bar charts; the sensory evaluation scores were converted into radar charts.

## 3 Results and discussion

### 3.1 Sensory evaluation

**FIGURE 1:**
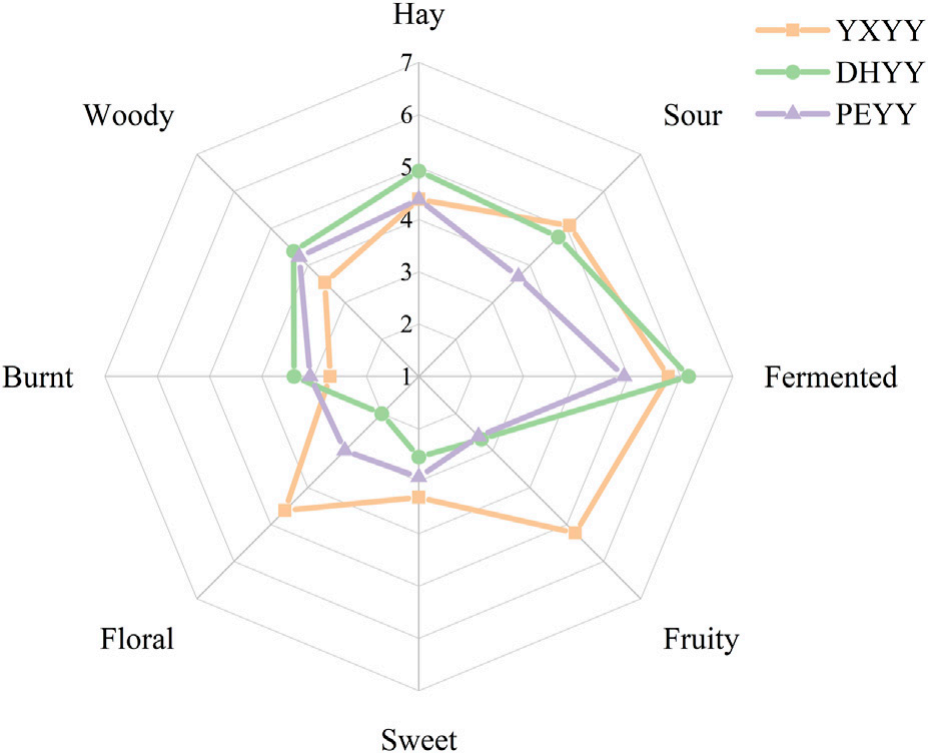
Sensory evaluation of aroma profile of cigar tobacco in three regions.

The sensory characteristics of the three cigar samples were obtained through sensory evaluation and the aroma profiles were plotted (Figure 1). Eight core sensory evaluation terms were identified, including hay, sour, fermented, fruity, sweet, floral, burnt and woody, to fully characterize the complex aroma attributes of cigar tobacco. The results showed that sour, fermented and hay aromas constituted the main aromas, and their mean values of perceived intensity were higher than those of the other aromas. Overall, the three cigar tobaccos showed significant differences in aroma profiles, with YXYY having the richest overall aroma profile, with greater intensity in sour, fruity and floral aromas, and weaker intensity in burnt, woody and hay aromas; DHYY showed sensory attributes similar to those of YXYY in sour and fermented aromas, and had higher intensity in hay and woody than that of YXYY, while its floral and sweet aromas were the weakest; PEYY had the weakest overall aroma; PEYY had the lowest overall aroma, with higher mean value of perceived intensity than that of other aromas. DHYY showed similar sensory attributes to YXYY in sour and fermentation aroma, and the intensity of hay and wood aroma was higher than that of YXYY, with the floral and sweet aroma being the weakest. In addition, the eight sensory attributes showed different degrees of differences among the three types of cigar tobaccos, with no significant differences in the three sensory attributes of hay, caramelized aroma and woody aroma. In terms of floral aroma, there is a big difference among the three types of cigar leaves, with YXYY having the strongest aroma, followed by PEYY, and DHYY having the weakest floral aroma. The intensity of sweet and fresh aroma was similar to that of floral aroma, with YXYY being the strongest, while for fruity aroma, DHYY and PEYY had no significant difference and weak aroma, while YXYY was more prominent in fruity aroma. In terms of sour and fermented aroma, YXYY and DHYY are similar in intensity and better than PEYY. In conclusion, YXYY has the richest aroma, followed by DHYY, and PEYY is weaker in overall aroma.

### 3.2 Cigar tobacco aroma active compounds

**FIGURE 2:**
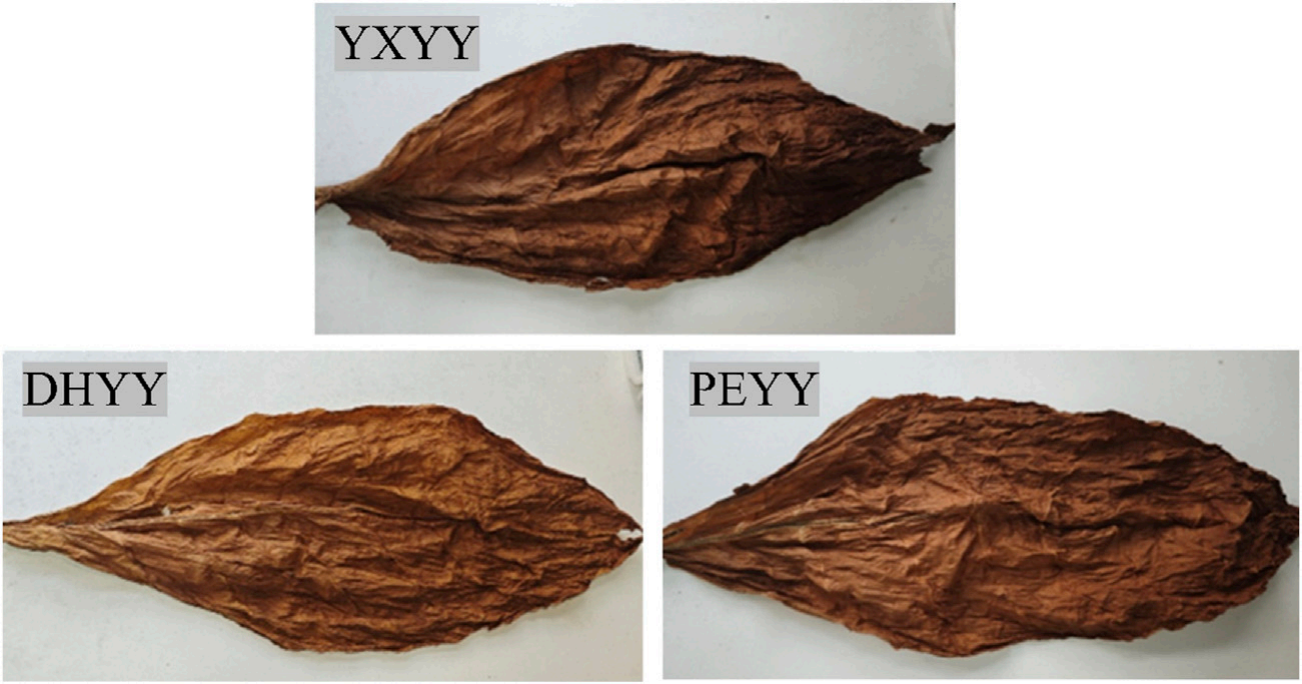
Appearance of three types of cigar tobacco leaves.

In this study, the SAFE method was used to extract the volatiles from the three types of cigar leaves shown in Figure 2. A total of 110 volatile compounds were detected, including 14 amides, 12 aromatic compounds, 8 acids, 4 olefins, 12 alcohols, 1 aldehyde, 18 ketones, 6 esters, 31 heterocyclic compounds, and 4 others, which were in 10 major groups. Among these volatile compounds, there were 92 volatile compounds in YXYY, 60 in DHYY and 68 in PEYY (Table 1). The number of volatile compounds in cigar filler tobacco was significantly higher than that in wrapper tobacco. This phenomenon may be attributed to the thicker and more compact cellular structure of cigarillo wrapper tobacco, which possesses the capacity to retain a greater quantity of oils and volatile compounds. The structural characteristics of the wrapper leaf, namely its thin structure, flexibility and uniform oil content, serve to limit the accumulation of aroma substances. This phenomenon may also be attributed to the fact that the primary breeding objective of the wrapper leaf is to enhance aroma intensity and complexity. The aubergine varieties, on the other hand, are more concerned with appearance (e.g., uniformity of color, absence of blemishes) (Huang et al., 2025), which leads to the natural differentiation of the chemical composition of the two varieties.

**TABLE 1:** Aroma compounds in YXYY, DHYY and PEYY.

No.	Compounds	CAS number	Method	DH	PE	YX
1	N-Methylformamide	123-39-7	MS/RI	√	√	√
2	Acetamide	60-35-5	MS/RI	√	√	√
3	N,N-Dimethylformamide	68-12-2	MS/RI	√	√	√
4	N,N-Dimethylacetamide	127-19-5	MS/RI	-	-	√
5	N-Methylbutyramide	1121-07-9	MS/RI	√	√	√
6	Isobutyramide	541-46-8	MS/RI	-	-	√
7	Ethylacetamide	77-67-8	MS/RI	√	√	√
8	2-Phenylacetamide	103-81-1	MS/RI	√	√	√
9	N-Methylnicotinamide	114-33-0	MS/RI	√	√	√
10	N,N-Dimethylcaproamide	14433-76-2	MS/RI	√	-	-
11	Dibenzylamine	103-49-1	MS/RI	-	√	-
12	2-Pyridinecarboxamide	1452-77-3	MS/RI	-	-	√
13	Isobutyramide	563-83-7	MS/RI	-	-	√
14	N-Methylacetamide	79-16-3	MS/RI	-	-	√
15	Toluene	108-88-3	MS/RI	√	√	√
16	m-Xylene	108-38-3	MS/RI	√	-	√
17	Styrene	100-42-5	MS/RI	-	-	√
18	Phenethyl alcohol	60-12-8	MS/RI	√	√	√
19	Phenol	108-95-2	MS/RI	-	-	√
20	2,4-Di-tert-butylphenol	96-76-4	MS/RI	√	√	√
21	Benzoic acid	65-85-0	MS/RI	√	√	√
22	Diisobutyl phthalate	84-69-5	MS/RI	√	√	√
23	Phenylacetic acid	103-82-2	MS/RI	√	√	√
24	Di-n-butyl phthalate	84-74-2	MS/RI	√	√	√
25	Benzaldehyde	100-52-7	MS/RI	√	√	-
26	Acetophenone	98-86-2	MS/RI	-	-	√
27	7,11,15-Trimethyl-3-methylidene-hexadec-1-ene	504-96-1	MS/RI	√	√	√
28	Cedrol	77-53-2	MS/RI	√	√	√
29	Cedrene	189-13-1	MS/RI	√	√	√
30	Gammarotene	3242-08-8	MS/RI	-	-	√
31	(E)-5-isopropyl-8-methylnona-6,8-dien-2-one	54868-48-3	MS/RI	√	√	√
32	3-Methyl-2-heptanone	2371-19-9	MS/RI	-	-	√
33	3-Hydroxy-2-butanone	513-86-0	MS/RI	-	-	√
34	4-Hydroxy-2-butanone	590-90-9	MS/RI	-	-	√
35	Isoforone	78-59-1	MS/RI	-	-	√
36	Zythone	502-69-2	MS/RI	√	√	√
37	4,7,9-Megastigmatrien-3-one A	38818-55-2	MS/RI	√	√	√
38	4,7,9-Megastigmatrien-3-one B	38818-55-2	MS/RI	√	√	√
39	4,7,9-Megastigmatrien-3-one C	38818-55-2	MS/RI	√	√	√
40	4,7,9-Megastigmatrien-3-one D	38818-55-2	MS/RI	√	√	√
41	3,5,5-Trimethyl-4-hydroxy-2-cyclohexen-1-one	14203-59-9	MS/RI	-	-	√
42	Farnesyl acetone	1117-52-8	MS/RI	√	√	√
43	2-(Formyloxy)-1-phenylacetone	55153-12-3	MS/RI	-	-	√
44	3-Oxo-α-ionol	34318-21-3	MS/RI	√	√	√
45	4-(3-Hydroxybutyl)-3,5,5-trimethylcyclohex-2-en-1-one	36151-02-7	MS/RI	√	-	√
46	6-Methyl-5-hepten-2-one	110-93-0	MS/RI	-	√	√
47	4-Oxo-isofurone	1125-21-9	MS/RI	-	√	-
48	Dihydrojasmone	1128-08-1	MS/RI	-	√	√
49	cis-3-Hexenol formate	33467-73-1	MS/RI	√	-	-
50	Methyl carbamate	598-55-0	MS/RI	-	-	√
51	Isooctyl acrylate	103-11-7	MS/RI	-	-	√
52	Methyl palmitate	112-39-0	MS/RI	√	√	√
53	2-Ethylhexyl acetate	103-09-3	MS/RI	-	-	√
54	Linalyl acetate	115-95-7	MS/RI	√	√	-
55	Nonanal	124-19-6	MS/RI	√	√	√
56	Acetic acid	64-19-7	MS/RI	√	√	√
57	Palmitic acid	57-10-3	MS/RI	√	√	√
58	Pentanoic acid	109-52-4	MS/RI	-	√	√
59	Nonanoic acid	112-05-0	MS/RI	-	√	-
60	Propionic acid	79-09-4	MS/RI	-	-	√
61	Isobutyric acid	79-31-2	MS/RI	-	-	√
62	Butyric acid	107-92-6	MS/RI	-	-	√
63	Isobutyric acid	503-74-2	MS/RI	-	-	√
64	Tertiary butanol	75-85-4	MS/RI	√	√	√
65	2-Methyl-3-buten-2-ol	115-18-4	MS/RI	√	√	√
66	2,3-Butanediol	513-85-9	MS/RI	√	√	√
67	Propylene glycol	57-55-6	MS/RI	√	√	-
68	Iso-santalol	25269-17-4	MS/RI	√	√	√
69	Phytol	150-86-7	MS/RI	√	√	√
70	2-Ethylhexanol	104-76-7	MS/RI	-	√	√
71	Sclareol	515-03-7	MS/RI	-	-	√
72	Linalool	78-70-6	MS/RI	-	√	-
73	Isophytol	505-32-8	MS/RI	-	√	-
74	Geranyl linalool	1113-21-9	MS/RI	-	√	√
75	Trans-nerolidol	40716-66-3	MS/RI	-	-	√
76	4,5-Dimethyl-2(3H)-furanone	6971-63-7	MS/RI	√	-	-
77	4-Methyl-5,6-dihydro-2-furanone	2381-87-5	MS/RI	√	-	√
78	5,6-Dihydro-6-pentyl-2H-furan-2-one	54814-64-1	MS/RI	√	√	√
79	N-Methylpyrrolidone	872-50-4	MS/RI	√	√	√
80	2,3-Dimethyl maleic anhydride	766-39-2	MS/RI	-	-	√
81	2-Pyrrolidone	616-45-5	MS/RI	√	-	√
82	2-Aziridine-2,3-dione	675-20-7	MS/RI	√	√	√
83	2-Pyridine methanol	586-98-1	MS/RI	√	-	-
84	Indole	120-72-9	MS/RI	√	√	√
85	3-Ethyl-4-methyl-pyrrole-2,5-dione	20189-42-8	MS/RI	-	-	-
86	4-Phenylpyridine	939-23-1	MS/RI	-	-	√
87	2,3-Dimethylpyrazine	5910-89-4	MS/RI	-	-	√
88	2,6-Dimethylpyrazine	108-50-9	MS/RI	-	√	-
89	2,3,5-Trimethylpyrazine	14667-55-1	MS/RI	-	-	√
90	1,5-Dimethyl-2-pyrrolecarbonitrile	56341-36-7	MS/RI	-	√	-
91	3-Acetylpyridine	350-03-8	MS/RI	-	√	√
92	1-Acetylpyrrolidine	4030-18-6	MS/RI	-	-	√
93	3-Phenylpyridine	1008–88-4	MS/RI	-	√	-
94	trans-4-Dimethylaminocinnamaldehyde	4854-85-7	MS/RI	-	√	-
95	N-Formylpiperidine	2591-86-8	MS/RI	-	-	√
96	Nicotine	54-11-5	MS/RI	√	√	√
97	Mescaline	532-12-7	MS/RI	√	√	√
98	β-Nicotyrine	487-19-4	MS/RI	√	√	√
99	Neonicotine (dehydro-neonicotine)	2743-90-0	MS/RI	√	√	√
100	2,3′-Bipyridine	581-50-0	MS/RI	√	√	√
101	Methylarsonic acid lactone	674-26-0	MS/RI	-	-	√
102	Cotinine	486-56-6	MS/RI	√	√	√
103	Dihydrokiwi lactone	17092-92-1	MS/RI	√	√	√
104	Perilla lactone	564-20-5	MS/RI	√	√	√
105	2,5-Dihydrothiophen	1708-32-3	MS/RI	-	√	-
106	(+/−)-β-Hydroxy-γ-butyrolactone	5469-16-9	MS/RI	-	√	√
107	Propylene glycol methyl ether	107-98-2	MS/RI	√	-	√
108	o-Methoxybenzylamine	90-04-0	MS/RI	-	-	√
109	4-[2,2,6-Trimethyl-7-oxabicyclo [4.1.0]hept-1-yl]-3-buten-2-one	23267-57-4	MS/RI	√	√	√
110	Dimethyl sulfoxide	67-68-5	MS/RI	√	√	√

^a^ –, not detected.

^b^ √, detected.

The objective of this study is to undertake a comprehensive analysis of the active components present in the aroma of tobacco leaves, with a view to comparing the differences in aroma compounds among the three types of tobacco leaves under consideration. The analytical tool employed was GC-O-MS, a technique that integrates the separation capabilities of gas chromatography with the recognition abilities of human olfaction. As illustrated in Table 2, a total of 43 types of aroma compounds were identified in the three types of tobaccos. Of these, 30 types were detected by olfactory analysis in YXYY, of which 25 were characterized by the standard. Similarly, 29 types of aroma compounds were detected by olfactory analysis in DHYY, of which 24 were characterized by the standard. Finally, 21 types of aroma compounds were detected by olfactory analysis in PEYY, of which 16 were characterized by the standard. Heterocyclic compounds are the most abundant and are the main aroma substances in cigar tobacco. The majority of the pyrazine heterocyclic compounds were characterized by an intense, nutty and roasted flavor profile. Alkaloids have been demonstrated to exert a significant influence on the aroma, aroma volume, odor and off-gas of tobacco. Subsequent to the initial stage, the presence of ketones becomes evident. These acid compounds are characterized by a predominance of green and fruity flavors. Megastigmatrienone, a significant carotenoid degradation product, has been shown to impart a prolonged sweet tobacco flavor (Slaghenaufi et al., 2016). The presence of acid compounds is frequently associated with the occurrence of a stimulating sour flavor. Aldehydes are predominantly characterized by their greenish hue and oleaginous properties. The unique aroma profile of tobacco is shaped by the interaction of different compounds, each of which possesses distinct characteristics in terms of aroma intensity and character.

**TABLE 2:** GC-O results for three types of tobacco.

No.	CAS	Compounds	Odor	Sample			Identification method
				YXYY	DHYY	PEYY	
1	108-50-9	2,6-Dimethylpyrazine	Roasted	-^a^	-	√^b^	MS/RI/O/STD
2	100-42-5	Styrene	Floral, Sweet	√	√	√	MS/RI/O/STD
3	110-93-0	6-Methyl-5-hepten-2-one	Fruity	√	√	√	MS/RI/O/STD
4	5910-89-4	2,3-Dimethylpyrazine	Nutty, roasted	√	-	-	MS/RI/O/STD
5	103-09-3	2-Ethylhexyl acetate	Sour	√	√	-	MS/RI/O/STD
6	14667-55-1	2,3,5-Trimethylpyrazine	Sauce, roasted	√	√	-	MS/RI/O/STD
7	64-19-7	Acetic acid	Sour	√	√	√	MS/RI/O/STD
8	79-09-4	Poropanoic acid	Stimulating Sour	√	-	-	MS/RI/O/STD
9	79-31-2	Isobutyric acid	Stimulating Sour	√	-	-	MS/RI/O/STD
10	98-86-2	Acetophenone	Floral	√	-	-	MS/RI/O/STD
11	107-92-6	Butyric acid	Sour	√	-	-	MS/RI/O/STD
12	100-52-7	Benzaldehyde	Almond	-	√	-	MS/RI/O/STD
13	513-85-9	2,3-Butanediol	Fruity	-	√	-	MS/RI/O/STD
14	503-74-2	Isovaleric acid	Sour	√	-	-	MS/RI/O/STD
15		Unknown	Sweat	√	√	-	O
16	105-43-1	3-Methylvaleric acid	Stimulating Sour Scent	√	-	-	MS/RI/O/STD
17	60-35-5	Acetamide	Animal scent	√	√	-	MS/RI/O/STD
18	54868-48-3	(E)-5-isopropyl-8-methylnona-6,8-dien-2-one	Carrot flavor, tea flavor	√	√	√	MS/RI/O/STD
19	350-03-8	3-Acetylpyridine	Sweet aroma, baking aroma	√	√	√	MS/RI/O/STD
20		Unknown	Fishy odor	-	√	-	O
21	17283-81-7	Dihydro-β-Ionone	Floral and fruity	-	√	-	MS/RI/O/STD
22	54-11-5	Nicotine	Tobacco	√	√	√	MS/RI/O
23	60-12-8	Phenethyl alcohol	Rose	√	√	-	MS/RI/O/STD
24	504-96-1	7,11,15-Trimethyl-3-methylidene-hexadec-1-ene	Green	√	√	√	MS/RI/O/STD
25	14901-07-6	Irisone	Fruit and Flower	√	√	√	MS/RI/O/STD
26	23267-57-4	β-Ionone epoxide	Sweet, floral	√	√	-	MS/RI/O
27		Unknown	Strong and stimulating odors	√	√	√	O
28	77-53-2	Cedrol	Woody, sweet	√	-	-	MS/RI/O/STD
29	502-69-2	Phytone	Woody	√	√	√	MS/RI/O/STD
30	38818-55-2	4,7,9-Megastigmatrien-3-one	Tobacco	-	√	√	MS/RI/O
31	1898-13-1	Cembrene	Floral and fruity	-	-	√	MS/RI/O
32	532-12-7	Myosmine	Ammonia, baking	√	√	√	MS/RI/O/STD
33	38818-55-2	4,7,9-Megastigmatrien-3-one	Tobacco	√	√	√	MS/RI/O
34	54814-64-1	Massoia lactone	Herbal	√	√	√	MS/RI/O/STD
35	38818-55-2	4,7,9-Megastigmatrien-3-one	Tobacco flavor	√	-	-	MS/RI/O
36	487-19-4	β-Nicotyrine	Tobacco	-	√	√	MS/RI/O/STD
37	17092-92-1	Dihydroactinidiolide	Oily, fruity, sweet	-	√	-	MS/RI/O/STD
38	65-85-0	Benzoic acid	Stimulating acidic aroma	-	√	√	MS/RI/O/STD
39	120-72-9	Indole	Fecal odor, floral	√	-	-	MS/RI/O/STD
40	581-50-0	2,3′-Bipyridine	Tobacco	√	√	√	MS/RI/O/STD
41	103-82-2	Phenylacetic acid	Floral, sweet	-	√	√	MS/RI/O/STD
42	150-86-7	Phytol	Floral, green	√	√	√	MS/RI/O/STD
43	34318-21-3	3-Oxo-α-ionol	Pungent	√	√	√	MS/RI/O

^a^ –, not detected.

^b^ √, detected.

**FIGURE 3:**
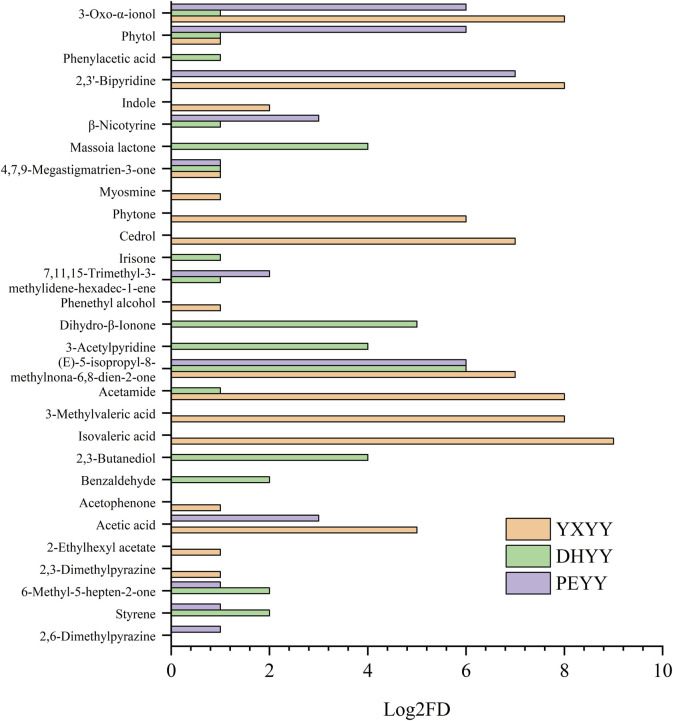
Number of species of aroma compounds in cigar tobacco.

As shown in Figure 3, the aroma compounds sniffed in YXYY included 9 heterocyclic compounds, 6 ketones, 6 acids, 3 aromatic compounds, 3 olefins, 1 alcohol, 1 ester, and 1 amide. 5 compounds could not be characterized as standards because they were not purchased, and 2 compounds with the odors of perspiration and strong irritation were sniffed, but could not be characterized because of the small peak areas. Two compounds with sweat odor and strong irritating odor were detected by sniffing, but could not be characterized due to the small peak area. Heterocyclic compounds were the most abundant and the main aroma substances in cigar tobacco, among which pyrazine heterocyclic compounds were mostly nutty and roasted. Alkaloids play a decisive role in the aroma, the amount of aroma, the taste and odor of the tobacco. Followed by ketones, acid compounds, ketones are mostly green, fruity flavor, acid compounds often bring stimulating acidic flavor, and aldehydes are mostly green, fatty flavor. These different compounds have their own characteristics in terms of aroma intensity and properties, and their interactions ultimately shape the unique aroma profile of the tobacco. 9 heterocyclic compounds, 7 ketones, 5 aromatic compounds, 2 alcohols, 1 ester, 1 acid, 3 olefins, and 1 amide were identified as aroma compounds in DHYY. Five compounds could not be characterized as standards for the time being because they were not purchased, and three compounds with sweaty, fishy and strong irritating odors were detected by olfaction, but they could not be characterized due to the masking of the small peak areas. Compared with YXYY, DHYY contained more heterocyclic compounds, which mainly brought the aroma of burnt, baked and breaded, followed by ketones and aromatic compounds, ketones were mainly green and fruity, and most of the aromatic compounds were floral and sweet.6 Heterocyclic compounds, 3 aromatic compounds, 7 ketones, 1 alcohol, 1 acid, 3 olefins and 3 olefin compounds were detected by olfaction. Compounds, 1 alcohol, 1 acid, and 3 olefins. Five compounds could not be characterized as standards for the time being because they were not available for purchase, and one compound with a strong and irritating odor was detected by sniffing, but could not be characterized because the peak area was small and was masked. The overall variety of compounds in PEYY was less than that of the first two types of tobacco, and the overall aroma was weaker, mainly consisting of ketones and heterocyclic compounds. The differences in the types of aroma compounds not only reflect the similarities but also the differences in the flavors of the three tobaccos, but also provide a certain basis for the subsequent research on the flavors of the three tobaccos.

### 3.3 Important aroma active compounds

**FIGURE 4:**
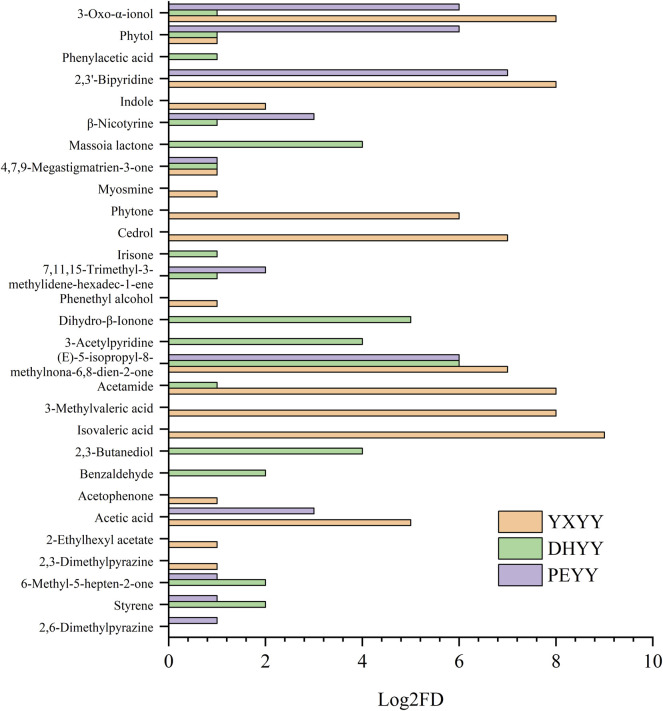
FD factors for aroma active compounds in cigar tobacco leaves from three regions.

In order to further explore the important aroma components in the tobacco leaves, AEDA analysis was performed, which is used to identify the compounds that contribute the most to the overall flavor by progressively diluting the sample and evaluating its aroma intensity. The results are shown in Figure 4 and Table 3, where 17, 16, and 11 aroma active compounds were identified as important aroma compounds for YXYY, DHYY and PEYY, respectively, which further demonstrate the diversity of aroma compounds of the three types of tobacco and their respective unique flavor profiles.

**TABLE 3:** Content of aroma compounds and OAV in three types of tobacco.

No.	CAS	Compounds	Calibration equations	*R* ^2^	Thresholds^b^(mg/kg)	Content (µg/g)			OAV		
						YXYY	DHYY	PEYY	YXYY	DHYY	PEYY
1	5910-89-4	2,3-Dimethylpyrazine	y = 0.8332x - 0.001	0.9953	0.8	0.049 ± 0	-^a^	-	<1	-	-
2	103-09-3	2-Ethylhexyl acetate	y = 1.4168x + 0.0053	0.9929	0.152	0.102 ± 0.003	-	-	<1	-	-
3	64-19-7	Acetic acid	y = 0.3695x - 0.0037	0.9962	1.74	144.328 ± 13.931	-	-	83	-	-
4	98-86-2	Acetophenone	y = 1.5946x + 0.0034	0.9933	1.95	0.066 ± 0.023	-	-	<1	-	-
5	503-74-2	Isovaleric acid	y = 0.5484x + 0.0352	0.9918	0.07	0.267 ± 0.16	-	-	4	-	-
6	105-43-1	3-Methylvaleric acid	y = 0.5194x - 0.0028	0.9952	0.163	0.708 ± 0.145	-	-	5	-	-
7	60-35-5	Acetamide	y = 0.5799x + 0.0011	0.9922	0.01	3.243 ± 0.083	1.795 ± 0.149	-	324	180	
8	54868-48-3	(E)-5-isopropyl-8-methylnona-6,8-dien-2-one	y = 0.0576x + 3E-06	0.9937	0.32	10.141 ± 6.381	4.839 ± 2.247	2.981 ± 0.86	32	3	9
9	60-12-8	Phenethyl alcohol	y = 1.2995x - 0.0539	0.9919	0.56	1.319 ± 0.02	-	-	2	-	-
10	77-53-2	Cedrol	y = 0.6132x - 0.0086	0.9915	21	0.843 ± 0.072	-	-	<1	-	-
11	502-69-2	Phytone	y = 1.9266x + 0.0061	0.996	-	1.222 ± 0.154	-	-	-	-	-
12	532-12-7	Myosmine	y = 0.8718x + 0.0728	0.9924	0.833	15.048 ± 1.684	-	-	18	-	-
13	120-72-9	Indole	y = 1.8148x + 0.0155	0.9912	28.45	0.118 ± 0	-	-	<1	-	-
14	581-50-0	2,3′-Bipyridine	y = 1.0737x + 0.0038	0.9997	456	22.727 ± 2.45	-	4.583 ± 1.719	<1	-	<1
15	150-86-7	Phytol	y = 0.665x - 0.0107	0.9992	2.61	2.613 ± 0.598	4.363 ± 0.426	-	1	2	-
16	100-42-5	Styrene	y = 1.7474x + 0.0142	0.9911	0.25	-	0.305 ± 0.038	0.162 ± 0.065	-	1	2
17	110-93-0	6-Methyl-5-hepten-2-one	y = 0.5667x + 0.0016	0.9936	0.08	-	0.081 ± 0.013	0.038 ± 0.011	-	1	<1
18	100-52-7	Benzaldehyde	y = 1.4101x + 0.0122	0.9924	0.085	-	0.088 ± 0.001	-	-	1	-
19	513-85-9	2,3-Butanediol	y = 0.5778x - 0.0404	0.9934	149.7	-	2.199 ± 0.355	-	-	<1	-
20	350-03-8	3-Acetylpyridine	y = 1.0926x - 0.0014	0.9975	0.5	-	0.279 ± 0.062	-	-	<1	-
21	504-96-1	7,11,15-Trimethyl-3-methylidene-hexadec-1-ene	y = 205.06x + 4.2375	0.9992	-	-	1.035 ± 0.187	0.477 ± 0.083	-	-	-
22	14901-07-6	Irisone	y = 0.8905x + 0.0009	0.9944	0.0084	-	0.101 ± 0.032	-	-	12	-
23	54814-64-1	Massoia lactone	y = 1.0007x + 0.185	0.9918	1.1	-	-	-	-	-	-
24	487-19-4	β-Nicotyrine	y = 412.74x - 0.0077	0.9991	1000	-	0.003 ± 0	-	-	<1	-
25	103-82-2	Phenylacetic acid	y = 0.8309x - 0.1452	0.9934	0.1	-	6.443 ± 2.448	-	-	65	-
26	108-50-9	2,6-Dimethylpyrazine	y = 0.5532x + 0.0178	0.9961	0.718	-	-	-	-	-	-
27	64-19-7	Acetic acid	y = 0.3695x - 0.0037	0.9962	1.74	-	-	88.26 ± 14.278	-	-	51
28	150-86-7	Phytol	y = 0.665x - 0.0107	0.9992	2.61	-	-	3.245 ± 0.557	-	-	1

^a^ –, not detected.

^b^ Odor thresholds reported in Ref. (ODOUR THRESHOLDS, compilations of odour threshold values in air, water and other media (Edition 2011)).

Three acids were found to have significant aroma contributions in YXYY, including isovaleric acid (sour odor, FD = 512), 3-methylpentanoic acid (sour odor, FD = 256), and acetic acid (sour aroma, FD = 32), which contributed a pungent sour aroma similar to the odor of perspiration in the tobacco. Acetamide (FD = 256), 2,3′-bipyridine (FD = 256), and 9-hydroxy-4,7-macrocarpalidin-3-one (FD = 256) also contributed to a greater extent to the aroma in YXYY and were considered to be its important aroma constituents, of which acetamide has a musty odor and is an amide, and most of the amides produce sensory effects such as musty odor, roughness and pungency (Leffingwell et al., 1972), which is mainly generated by amide groups at low temperatures and by amino groups at high temperatures, and it is hypothesized that it may be generated by the breakage of peptides formed by polymerization of asparagine (Hao et al., 2013), in addition, acetamide can bind to one bitter taste receptor protein, which may have a modifying effect on the bitter taste during vaping (Li et al., 2024); 2,3′-bipyridine is a secondary alkaloid in tobacco (Guo et al., 2022), which shares a similar structural and chemical properties to nicotine, presenting a tobacco flavor, and previous studies have also shown (Qin, 2019) that 2,3′-bipyridine has a significant potentiating effect on tobacco flavor and can suppress the irritation of tobacco to a certain extent; 9-hydroxy-4,7-macrogalladiene-3-one belongs to the degradation products of carotenoids, and possesses a pungent aroma. Other important aroma-active compounds found in YXYY: lycopene (FD = 128), which has a carrot-like aroma and a green, tea aroma, is produced by the degradation of cedratrienes, and is also the main aroma substance of white-ribbed cigarettes (Si et al., 2021), and its degradation product, lycopene acid, and its esters have a sweet and sour scent, which plays an important role in the aroma and aroma quality of cigarettes. Cedar brain (FD = 128) belongs to terpenoids, which is a naturally occurring sesquiterpene enol with a typical woody, medicinal aroma, and is widely found in tea (Cai et al., 2020) and medicinal herbs (Balaban-Uçar and Gönültaş, 2019). These aroma actives mainly provided sour, fermented, hay and fruity aromas, which were in high agreement with the sensory evaluation terms obtained from the screening and discussion of the sensory evaluation panel.

Of the 17 important aroma active compounds in DHYY, 5 were the same as in YXYY, with the largest FD (64) being the carrot and tea aroma of cannabinone. In addition, β-dihydroviologenone (FD = 32) with floral and fruity aroma, 2,3-butanediol (FD = 16) with fruity aroma, 3-acetylpyridine (FD = 16) with baking aroma, and 5,6-dihydro-6-pentyl-2H-pyran-2-one (FD = 16) with herbal aroma were also considered important aroma compounds with the same intensity in the DHYY. Among them, β-dihydroviolanone and β-violanone belong to terpenoids, which are produced by the degradation of carotenoids (Wang et al., 2020), and provide light floral and fruity aroma in tobacco, while 3-acetylpyridine is a product of the Maillard reaction in tobacco, with nutty and roasted aroma.

Of the 11 important aroma active compounds in PEYY, 10 were the same as in DHYY, but the intensity of the aroma was different. Unlike DHYY, 2,6-dimethylpyrazine (FD = 2), which is characterized by baking and nutty aroma, was also found in PEYY, which may be the key substance that distinguishes the overall aroma of PEYY from that of DHYY. In addition, both 2,3′-bipyridine (FD = 128) and phytol (FD = 64) were found to have stronger aroma intensities in PEYY than in DHYY, where phytol, a diterpene alcohol with floral and green aroma, is one of the more important volatiles found in cigar tobacco (Wu et al., 2024), and also provides a light grassy aroma in tea (Malongane et al., 2020).

### 3.4 Quantitation of aroma compounds and OAV evaluation

**FIGURE 5:**
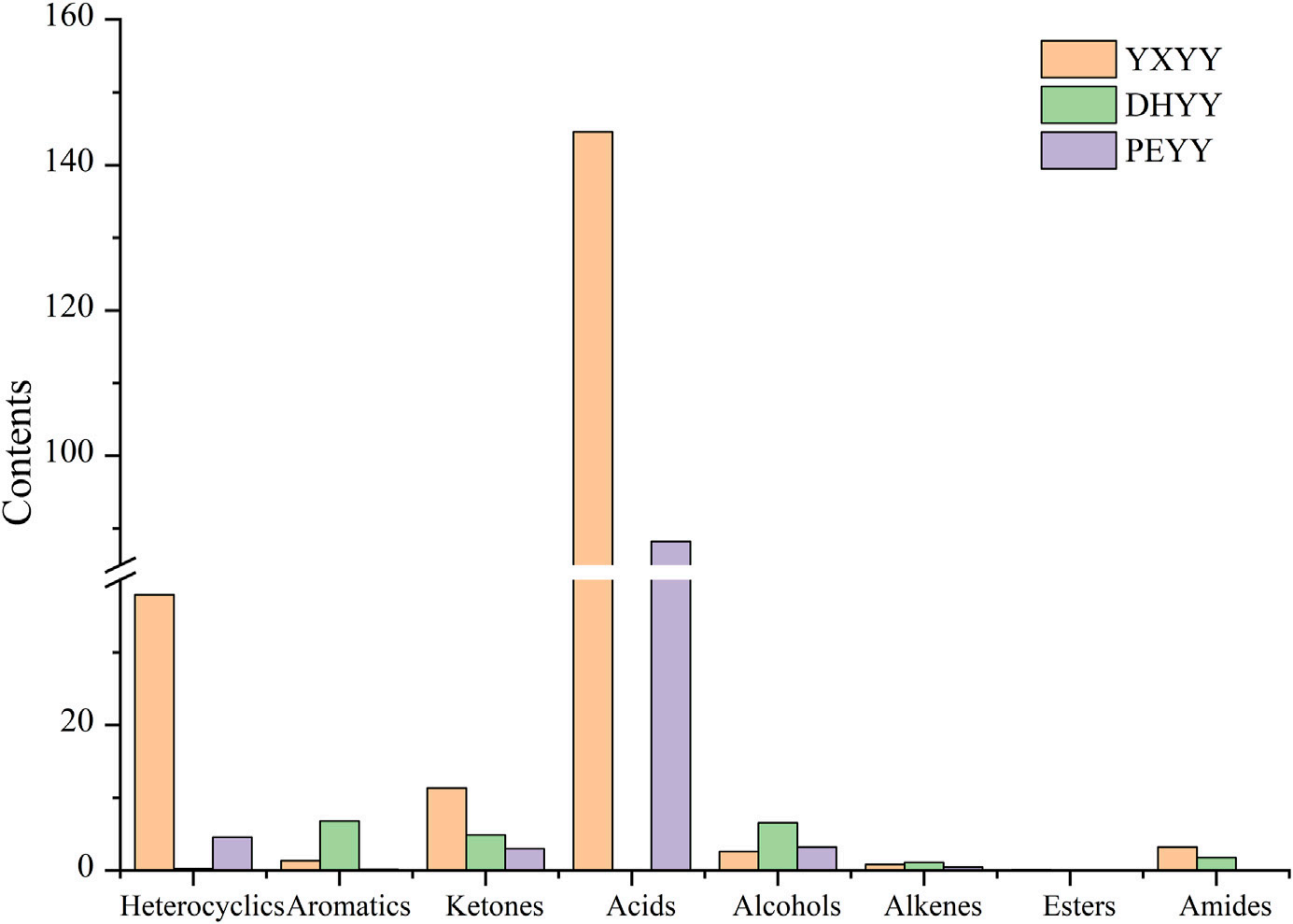
Content of aroma compounds in cigar tobacco.

In order to further identify the key aroma compounds in tobacco, the important aroma active compounds (FD ≥ 2) in three kinds of tobacco were accurately quantified by the internal standard curve method. The established standard curves are shown in Figure 5 and Table 3. The standard curves were linear (R2≥0.99) and the OAV values were calculated according to the threshold values.

The highest contents of acid compounds were found in YXYY and PEYY, which gave the unique sour aroma to the tobaccos, and the highest contents of aromatic compounds were found in DHYY. The highest content of aromatic compounds was found in DHYY. In addition, the content of heterocyclic compounds in YXYY was also higher at 37.942 μg/g, which provided unique roasted and burnt aroma to the tobacco. The quantitative results showed that the top two most abundant compounds in YXYY and PEYY were the same, which were acetic acid and 2,3′-bipyridine. The contents of these two substances were higher in YXYY than in PEYY, with the highest contents of acetic acid reaching 144.328 μg/g and 88.26 μg/g in YXYY and PEYY, respectively; followed by 2,3′-bipyridine, but the contents of these two substances varied greatly between the two kinds of tobacco, with 22.727 μg/g in YXYY and 4.583 μg/g in PEYY, respectively. In addition, the compounds in YXYY were mescaline (15.048 μg/g) and (E)-5-isopropyl-8-methylnona-6,8-dien-2-one (10.141 μg/g), while the concentrations of the other compounds were all below 10 μg/g. The compounds that were more abundant in PEYY were phytol (3.245 μg/g) and (E)-5-isopropyl-8-methylnona-6,8-dien-2-one (2.981 μg/g), etc. The compounds that were more abundant in DHYY were phenylacetic acid (6.443 μg/g), (E)-5-isopropyl-8-methylnona-6,8-dien-2-one (4.839 μg/g), phytol (4.363 μg/g) and 2,3-butanediol (2.199 μg/g). Although some of the aroma active compounds were found in relatively small amounts in the tobacco, they had large FD factors, indicating that they still have the potential to contribute significantly to the overall tobacco aroma.

In general, aroma compounds with OAV values greater than or equal to 1 are considered as key aroma active compounds that can affect the overall aroma of the samples. The OAVs of 15 aroma compounds in YXYY, 12 aroma compounds in DHYY and 8 aroma compounds in PEYY were calculated for the three cigar tobacco samples, and the OAVs of 8, 8, and 4 aroma compounds with OAV ≥1 in YXYY, DHYY and PEYY, respectively, which were considered to be the key compounds of the tobacco and had important contributions to the overall aroma of the tobacco. These aroma compounds are considered as key aroma compounds in tobacco, which have important contributions to the overall aroma of tobacco. Among the eight key aroma active compounds with OAV≥1 in YXYY, acetamide and acetic acid had higher OAVs of 324 and 83, respectively. Among them, although the content of acetamide was low (3.243 μg/g), it made an important contribution to the fermentation aroma of tobacco due to its low threshold value (0.01); followed by acetic acid, whose content was the highest and conferred a sour aroma to the tobacco, and they made a They contributed more to the overall aroma of the tobacco. The OAV of 32 for (E)-5-isopropyl-8-methylnona-6,8-dien-2-one, with carrot and tea aroma, gave the characteristic aroma of fruity and sweet aroma to the tobacco (Jiang et al., 2024), which is an important component of the unique flavor of YXYY. The OAV results showed that the aroma compounds with high OAV in the tobacco samples mainly contributed to the aroma characteristics of fermentation, sour, fruity and sweet aroma. Other aroma active components contributed to the aroma attributes of YXYY hay, woody, floral and burnt aroma. Among the eight key aroma compounds of DHYY, the compound with the larger OAV was acetamide, as in YXYY, which might be the key compound contributing to the greater intensity of fermentation aroma in YXYY and DHYY than in PEYY. The second compounds were phenylacetic acid and irisone, which made important contributions to the study of the characteristic flavor components of DHYY’s floral cigar tobacco and smoke, such as 22 aroma and sweet and light aroma. In PEYY, there were no compounds with OAV greater than 100, which differed greatly from other tobaccos, and the two compounds with larger OAV were acetic acid (51) and (E)-5-isopropyl-8-methylnona-6,8-dien-2-one (10), which also presented important aromas in YXYY. In contrast to YXYY, the overall aroma profiles of DHYY and PEYY were closer, and six key aroma compounds were common to both tobaccos, namely styrene (floral, sweet), 6-methyl-5 hepten-2-one (fruity), (E)-5-isopropyl-8-methylnona-6,8-dien-2-one (carrot, tea), 7,11,15-Trimethyl-3-methylidene-hexadec-1-ene (green), β-Nicotyrine (tobacco), and phytol (green, fruity). They may be more important common aroma compounds in cigar-coated tobacco, and together they constitute the key substances for the unique aroma of the tobacco.

### 3.5 Aroma recombination and omission experiments

**FIGURE 6:**
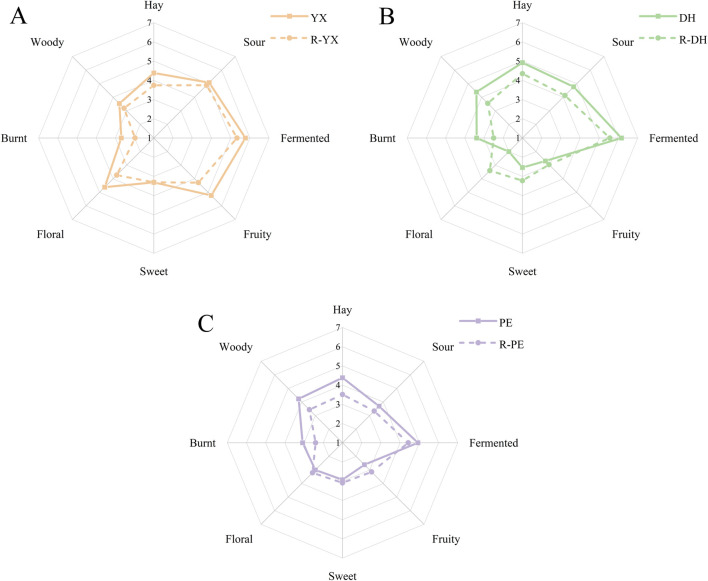
Sensory evaluation radar chart of the recombinant model and original tobacco samples (**(A)**, YX: YX cigar tobacco leaves, R-YX: recombinant YX cigar tobacco leaves; **(B)**, DH: DH cigar tobacco leaves, R-DH: recombinant DH cigar tobacco leaves; **(C)**, PE: PE cigar tobacco leaves, R-PE: recombinant PE cigar tobacco leaves).

The results of the sensory evaluation of the three tobaccos and their recombinant models are shown in Figure 6. Through the comparative evaluation, it was found that the aroma profiles of the recombinant models matched well with the aroma profiles of the original samples of YXYY, DHYY, and PEYY, which effectively simulated the flavor profiles of the tobaccos, and it was also verified that the key aroma compounds were basically correct. However, there are some differences between them, for example, the overall aroma intensity of the recombinant model of YXYY was lower than that of the original samples, probably because the screening criterion of the recombinant samples was OAV ≥1, so some aroma active compounds, such as acetophenone, indole and other compounds, were not added to the recombinant model (OAV <1), which resulted in the low intensity of the floral and fruity aroma; and also because the standardized compounds such as macadamia trienone and megadamia trienone were not added to the recombinant model (OAV <1). It may also be due to the absence of standard compounds such as meglumine trienone, which could not be calculated and not added to the recombinant model, resulting in low hay and burnt aroma, which needs to be further verified in subsequent studies to determine the criticality of the aroma active compounds in the tobacco leaf. In the DHYY recombination model, the fermented and fruity aroma scores were very close to each other with high similarity, while the floral, sweet and fruity aromas were stronger than the original samples, which might have affected the overall performance of the fermented aroma to a certain extent, making the fermented aroma of the recombined samples slightly inferior to that of the actual samples, and it is reasonable to speculate that weakening the floral and fruity aroma intensities appropriately might further emphasize the fermented aroma. In the PEYY recombinant model, the intensity of hay, wood, and burnt aroma was slightly lower than that of the original sample, which may be due to the lack of 2,6-dimethylpyrazine, diene nicotine, and 2,3′-bipyridine (OAV <1) compounds that have the characteristic aroma of tobacco.

In order to further verify the degree of contribution of each aroma active compound to the overall flavor of tobacco, triangulation tests were done by missing a single aroma active substance and two complete recombinant models respectively, and the correctness and significance of the judgments are shown in Table 4. In YXYY, eight groups of missing models were formulated, and the results showed that the recombinant model with phytol omitted exhibited a highly significant difference in the aroma of the recombinant model with a complete recombinant model (α ≤ 0.001). The results showed that the recombinant model omitting phytol exhibited a highly significant difference in aroma from the full recombinant model (α ≤ 0.001), contributing herbaceous and green aromas, which were the main contributors to the aroma profile in YXYY. In addition models missing acetic acid (α ≤ 0.01), 3-methylpentanoic acid (α ≤ 0.05), isovaleric acid (α ≤ 0.05), and kauranone (α ≤ 0.05), respectively, were also significant, suggesting that these substances also play a large role in the contribution of the aroma of YXYY. Differently, the model for the absence of styrene (α ≤ 0.001) in DHYY was more significant, presenting a floral and sweet aroma, and kauranone (α ≤ 0.05), β-violetone (α ≤ 0.05), and phytochemicals (α ≤ 0.05) were also important in DHYY, contributing significantly to the overall aroma profile of DHYY. The absence of acetic acid (α ≤ 0.01), styrene (α ≤ 0.05), and phytol (α ≤ 0.05) in PEYY was modeled to be significant, showing that they are important aroma compounds in PEYY, and the removal of the cannabinoid compounds did not result in a significant difference in the system’s aroma properties (α > 0.05), suggesting that they make a small contribution to the overall aroma of PEYY, and are not considered to be key aroma components.

**TABLE 4:** Results of the omission experiment for key aroma substances (OAV ≥1) in cigar tobaccos.

	YXYY			DHYY			PEYY	
No.	Compounds	Significance	No.	Compounds	Significance	No.	Compounds	Significance
1	Phytol	***	1	Styrene	***	1	Acetic acid	**
2	Acetic acid	**	2	(E)-5-isopropyl-8-methylnona-6,8-dien-2-one	*	2	Styrene	*
3	Isovaleric acid	*	3	β-Nicotyrine	*	3	Phytol	*
4	3-Methylvaleric acid	*	4	Phytol	*	4	(E)-5-isopropyl-8-methylnona-6,8-dien-2-one	-
5	(E)-5-isopropyl-8-methylnona-6,8-dien-2-one	*	5	6-Methyl-5-hepten-2-one	-			
6	Acetamide	-	6	Benzaldehyde	-			
7	Phenethyl alcohol	-	7	Acetamide	-			
8	Myosmine	-	8	Phenylacetic acid	-			

*, significant (α ≤ 0.05); **, highly significant (α ≤ 0.01); ***, very highly significant (α ≤ 0.001); -, nonsignificant.

## 4 Conclusion

In this study, the volatile compounds in cigar tobacco leaves from three regions were systematically investigated using molecular sensory science methods. Using GC-O-MS, 30 aroma compounds in YXYY, 29 aroma compounds in DHYY and 21 aroma compounds in PEYY were detected, and the key aroma active compounds with FD ≥ 2 and OAV ≥1 were selected, of which there were 8, 8, and 4 in YXYY, DHYY and PEYY, respectively. Through recombination and omission experiments, the key aroma actives were further identified as phytol, acetic acid, isovaleric acid, 3-methylpentanoic acid and (E)-5-isopropyl-8-methylnona-6,8-dien-2-one in YXYY, styrene, (E)-5-isopropyl-8-methylnona-6,8-dien-2-one, irisone, and phytol in DHYY, and acetic acid, styrene, and phytol in PEYY. Phytol was the key aroma compound in all samples, and the other substances were differential aroma markers in the cigars. This study provided a comprehensive aroma profile of cigar tobacco from different origins and yielded key aroma substances that provide a foundation for the aroma of cigar tobacco. Future research will focus on elucidating the pathways and influences of these key aroma compounds in order to tailor the aroma of cigar tobacco.

## Data Availability

The original contributions presented in the study are included in the article/supplementary material, further inquiries can be directed to the corresponding authors.
